# Direct Measurements of Smartphone Screen-Time: Relationships with Demographics and Sleep

**DOI:** 10.1371/journal.pone.0165331

**Published:** 2016-11-09

**Authors:** Matthew A. Christensen, Laura Bettencourt, Leanne Kaye, Sai T. Moturu, Kaylin T. Nguyen, Jeffrey E. Olgin, Mark J. Pletcher, Gregory M. Marcus

**Affiliations:** 1 Division of Cardiology, Department of Medicine, University of California San Francisco, San Francisco, California, United States of America; 2 Department of Epidemiology and Biostatistics, University of California San Francisco, San Francisco, California, United States of America; 3 Ginger.io Incorporated, San Francisco, California, United States of America; University of Rome Tor Vergata, ITALY

## Abstract

**Background:**

Smartphones are increasingly integrated into everyday life, but frequency of use has not yet been objectively measured and compared to demographics, health information, and in particular, sleep quality.

**Aims:**

The aim of this study was to characterize smartphone use by measuring screen-time directly, determine factors that are associated with increased screen-time, and to test the hypothesis that increased screen-time is associated with poor sleep.

**Methods:**

We performed a cross-sectional analysis in a subset of 653 participants enrolled in the Health eHeart Study, an internet-based longitudinal cohort study open to any interested adult (≥ 18 years). Smartphone screen-time (the number of minutes in each hour the screen was on) was measured continuously via smartphone application. For each participant, total and average screen-time were computed over 30-day windows. Average screen-time specifically during self-reported bedtime hours and sleeping period was also computed. Demographics, medical information, and sleep habits (Pittsburgh Sleep Quality Index–PSQI) were obtained by survey. Linear regression was used to obtain effect estimates.

**Results:**

Total screen-time over 30 days was a median 38.4 hours (IQR 21.4 to 61.3) and average screen-time over 30 days was a median 3.7 minutes per hour (IQR 2.2 to 5.5). Younger age, self-reported race/ethnicity of Black and "Other" were associated with longer average screen-time after adjustment for potential confounders. Longer average screen-time was associated with shorter sleep duration and worse sleep-efficiency. Longer average screen-times during bedtime and the sleeping period were associated with poor sleep quality, decreased sleep efficiency, and longer sleep onset latency.

**Conclusions:**

These findings on actual smartphone screen-time build upon prior work based on self-report and confirm that adults spend a substantial amount of time using their smartphones. Screen-time differs across age and race, but is similar across socio-economic strata suggesting that cultural factors may drive smartphone use. Screen-time is associated with poor sleep. These findings cannot support conclusions on causation. Effect-cause remains a possibility: poor sleep may lead to increased screen-time. However, exposure to smartphone screens, particularly around bedtime, may negatively impact sleep.

## Introduction

Smartphones are increasingly integrated into everyday life [1]. At the same time, the prevalence of insomnia and sleep deprivation have risen [2]. Poor sleep (too little or too much, and poor quality) has been shown to be a risk factor for obesity, diabetes, cardiovascular disease, depression, and overall mortality [3,4]. Light in the blue spectrum, such as light produced from a smartphone, can suppress production of melatonin, leading to decreased drowsiness, difficulty initiating sleep, and non-restorative sleep [5,6]. In addition, engrossing activities during smartphone use may result in stimulation that is counter-productive to sleep preparation. Limiting the use of TV and computers near bedtime is commonly recommended as an important part of good sleep hygiene [7], but direct measurements of “screen-time” in native (or home) environments have not previously been available.

We sought to leverage data from the Health eHeart Study to (1) characterize the average amount and distribution of smartphone screen-time measured directly in a national cohort, (2) identify factors associated with increased screen-time, and (3) test the *a priori* hypothesis that increased screen-time is associated with decreased sleep quality.

## Methods

### Study Design

The Health eHeart Study (www.health-eheartstudy.org) is an internet-based prospective cohort that began enrolling any interested adult age ≥ 18 years with an active email address on March 8th, 2013. Participants were recruited via online and social media advertisements, email campaigns with research and advocacy organizations, and directly in person at University of California, San Francisco medical clinics. Consent was obtained electronically through the study website. We initially performed comparisons between prospectively collected screen-time and baseline demographics, medical history, and health behaviors in a hypothesis free manner. We then focused on participants who had completed a baseline sleep survey to specifically test the hypothesis that more screen-time would be associated with reduced sleep quality. The study was approved by the University of California, San Francisco Committee on Human Research.

### Measurement of Smartphone Screen-time

On September 1, 2014, participants with smartphones were invited to download a mobile application (“app”) developed by Ginger.io (San Francisco, CA); this mobile app was capable of recording screen-time on Android-based smartphones. Once downloaded, the app automatically operated in the background without disrupting normal use of the smartphone. The app recorded screen-time continuously as the number of minutes in each hour that the screen was turned on as long as the smartphone was not in “airplane mode” and the app was not actively turned off. This screen-time measure was stored locally on the smartphone and transmitted over the Internet to the study database daily.

Screen-time data was collected on existing and new participants between September 1, 2014 and September 30, 2015. The amount of missing data (due to phone-off, airplane mode, or app-off) in 30-day rolling windows was computed and the windows with the minimum level of missing data were identified and selected for analysis. In the event of ties, the earliest window with the minimum amount of missing data for each participant was selected for analysis. Participants with no screen-time data (100% missing data) or less than 30 days of observation were excluded. Within the selected 30-day window, the overall average screen-time (averaged over all hours in 30 days) and the hourly average screen-time (during each hour of the day, averaged over 30 days) were computed for each participant.

### Ascertainment of Demographic and Medical Information

Participants provided baseline personal, demographic, and medical information via several online surveys once during their initial “eVisit.” Race and ethnicity were combined into a single mutually exclusive covariate (“race/ethnicity”), where Hispanic ethnicity, if present, took precedence over selected race. Self-reported height and weight were used to calculate body mass index (BMI). The validated Patient Health Questionnaire Overview (PHQ-9) was used to assess mood [8]. The validated International Physical Activity Questionnaire (IPAQ) was used to assess physical activity [9]. The validated Pittsburg Sleep Quality Index (PSQI) was used to assess sleep duration, quality, and the sleeping period (bedtime and wake-up time) [10,11]. The total PSQI score and component sub-scores were analyzed as continuous variables and “poor sleep” was defined as dichotomous variable by PSQI total > 5, per instrument protocol [12].

### Statistical Analyses

Continuous variables with a normal distribution are presented as means ± standard deviations (SDs) and were compared with student’s t-tests. Non-normally distributed continuous variables are presented as medians and interquartile ranges (IQR) and were compared with the Wilcoxon rank-sum test. Categorical variables are presented as counts and percentages of the population and were compared with chi-squared tests.

Associations between average screen-time and baseline demographics, medical history, and behaviors were assessed first in a bivariate manner using linear regression models. Variables that were associated with average screen-time with two-tailed p < 0.10 in the bivariate analyses were retained in a single multivariate linear regression model where significance was considered at the standard two-tailed p < 0.05 level. Variables that remained statistically significantly associated with screen-time in the multivariate model were interpreted as independent predictors of screen-time. Variables that were no longer associated with screen-time in the multivariable model were considered to have been confounded by other variables, and were interpreted as not being key independent predictors. A sensitivity analysis was performed by restricting to those with complete screen-time data (no missing data) over the 30-day window.

Hypothesis-driven analyses related to screen-time and sleep were restricted to participants that completed the PSQI. Age, sex, and a history of sleep apnea were *a priori* “forced” into adjusted linear regression models along with other covariates that were significant (p<0.05) in the hypothesis-free multivariate model. Based on *a priori* hypotheses, select measures from the PSQI were compared to the average screen-time in the hours near self-reported bedtime (1 hour before participant-reported bedtime, the hour of participant-reported bedtime, 1 hour after the participant-reported bedtime) and during the sleep period (bedtime hour to wake-up hour) in a subgroup with no missing screen data. Statistical analyses were performed using STATA 13 (College Station, TX). Two tailed p values <0.05 were considered statistically significant.

## Results

### Participants

**Fig 1:**
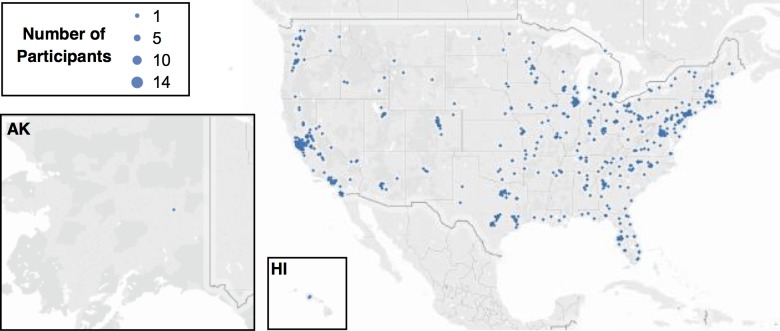
Geographical Distribution of Participants in the United States. Abbreviations: AK, Alaska; HI, Hawaii. Dots represent the number of participants that resided in the zip-code corresponding to the placement on the map. All 50 states were represented and 147 (23%) resided in California. Created with Tableau Software (www.tableau.com) and published with permission of the company (S1 File). The U.S. map was used under a CC BY-SA copyright from OpenStreetMap contributors (www.openstreetmap.org/copyright).

Among 23,187 Health eHeart participants enrolled by September 1, 2015, there were 3,566 that had downloaded the Ginger.io app of which 761 had Android-based phones that enabled capture of screen-time. Of these, 653 completed the core surveys in eVisit 1, had the app downloaded for at least 30 days, and had at least some (i.e., not 100% missing) screen-time data. Participants represented all 50 U.S. states and 147 (23%) resided in California (Fig 1). The baseline characteristics of the study population are presented in Table 1. Those with an average screen-time above the population median tended to be younger, female, Black, Hispanic, or “other” race/ethnicity, and have a higher PHQ-9 depression score. A sensitivity analyses restricted to a subgroup with 0% missing screen data (n = 292) revealed similar results (data not shown).

**Table 1:** Baseline Characteristics by Average Screen-Time.

Characteristics^a^		< median average screen-time (N = 325)^b^	≥ median average screen-time (N = 328)	P value^c^
Age, mean ± SD, years		52.2 ± 12.7	44.2 ± 11.9	< .001
Male sex, n (%)		96 (30%)	65 (20%)	.004
BMI, median (IQR), kg/m^2^		28.7 (24.0 to 33.5)	29.0 (24.1 to 34.5)	.67
Race/Ethnicity, n (%)				
	White	263 (82%)	224 (69%)	
	Black	16 (5%)	36 (11%)	
	Asian/Pacific-Islander	15 (5%)	12 (4%)	< .001
	Hispanic	15 (5%)	28 (9%)	
	Other	12 (4%)	24 (7%)	
Income, n (%), $ / year^d^				
	Less than 10,000	7 (2%)	16 (5%)	
	10,000–49,999	69 (22%)	89 (28%)	
	50,000–99,999	104 (33%)	100 (31%)	.12
	100,000–149,999	58 (18%)	45 (14%)	
	150,000 or more	54 (17%)	45 (14%)	
	Don’t know or decline	25 (8%)	27 (8%)	
Education, n (%)^c^				
	High school or less	9 (3%)	18 (6%)	
	Some college	95 (30%)	91 (28%)	
	Bachelor’s degree	88 (28%)	101 (31%)	.28
	Postgraduate	117 (37%)	107 (33%)	
	Don’t know or decline	8 (3%)	5 (2%)	
Alcoholic drinks / week, median (IQR)		3 (0 to 6)	2 (0 to 6)	.69
Smoking, n (%)				
	Never	200 (63%)	220 (69%)	
	Past	103 (33%)	83 (26%)	.14
	Current	13 (4%)	18 (6%)	
PHQ-9 depression score, median (IQR)		3 (1 to 6)	4 (2 to 8)	.002
IPAQ Activity level, n (%)				
	Low	1 (1%)	5 (4%)	
	Medium	40 (31%)	40 (29%)	.28
	High	90 (69%)	91 (67%)	
Diagnoses, n (%)				
	Atrial fibrillation	32 (10%)	17 (5%)	.02
	CAD	32 (10%)	37 (12%)	.59
	CHF	17 (5%)	17 (5%)	.95
	Diabetes	38 (12%)	33 (10%)	.48
	Hyperlipidemia	167 (53%)	125 (39%)	< .001
	HTN	138 (44%)	117 (37%)	.06
	Obstructive sleep apnea	50 (16%)	54 (17%)	.78
**Subset with sleep survey**		**(N = 78)**	**(N = 58)**	
PSQI total, median (IQR)		4 (3 to 7)	5 (3 to 8)	.33
Poor sleep (PSQI total > 5), n (%)		27 (35%)	24 (41%)	.42

Abbreviations: IQR, interquartile range; SD, standard deviation; PHQ-9, patient health questionnaire; IPAQ, international physical activity questionnaire; CAD, coronary artery disease; CHF, congestive heart failure; HTN, hypertension; PSQI, Pittsburgh sleep quality index.

^a^ All 653 participants provided age. There number of participants with data for each covariate were: male sex, 645 (99%); BMI, 590 (90%); race/ethnicity, 645 (99%); income and education each, 639 (98%); alcohol, 394 (60%); smoking, 637 (98%); PHQ-9, 631 (97%); IPAQ, 267 (42%); atrial fibrillation, 624 (96%); CAD, 635 (97%); CHF, 636 (97%); diabetes, 636 (97%); hyperlipidemia, 633 (97%); HTN, 635 (97%); Obstructive sleep apnea, 616 (94%).

^b^ The population median of individual average screen-times was 3.7 (IQR 2.2–5.5) minutes / hour

^c^ Student T-test’s were used to compare normally distributed continuous variables, Wilcoxon rank-sum tests were used for non-normally distributed continuous variables, chi-square tests were used for categorical variables.

^d^ Income (U.S. dollars) and education where both ascertained as 9-level ordinal categorical variables. Categories were condensed for presentation.

**Fig 2:**
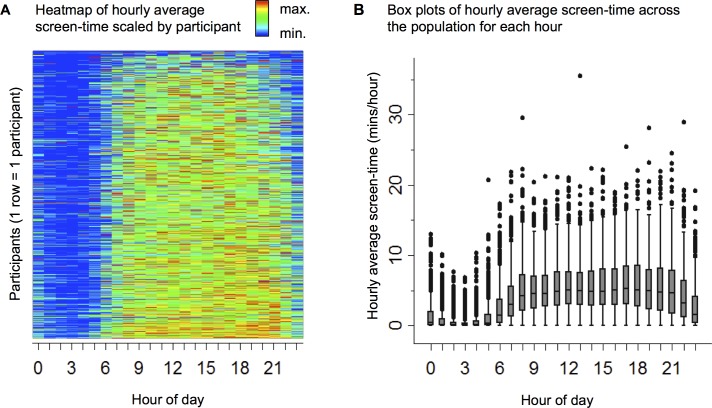
Distribution of Screen-Time Over the Day (Hourly Average Screen-Time). (A) Hourly average screen-time scaled to the maximum within each participant: blue = minimum; red = maximum. Each horizontal line represents data for one participant across 24 hours in a day. (B) Box plots of population summary statistics of hourly average screen-time. Horizontal line within box = median, boxes = IQR, whiskers = 1.5 interquartile range (IQR), dots = outliers.

Within the selected 30-day windows, the app recorded screen-time for a median 29.9 (IQR 27.3 to 30.0) days, during which total screen-time was a median 38.4 (IQR 21.4 to 61.3) hours and average screen-time was a median 3.7 (IQR 2.2 to 5.5) minutes per hour. This average screen-time is equivalent to 1 hour and 29 minutes (IQR 53 minutes to 2 hours and 12 minutes) per day. The relative distribution of screen-time within participants and hourly average screen-time across the population are shown in Fig 2.

### Associations with Screen-Time

**Fig 3:**
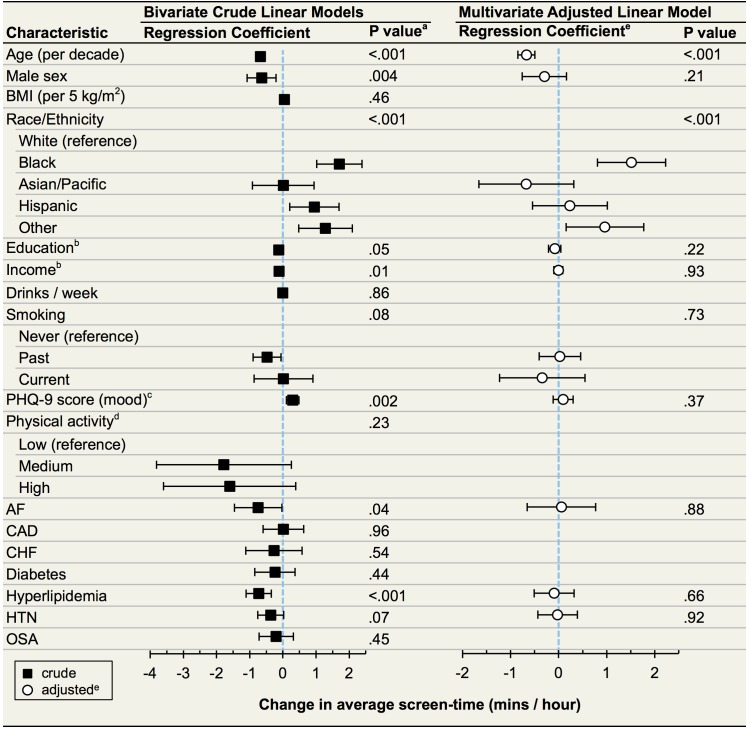
Associations Between Baseline Survey Data and Average Screen-Time (N = 653). Abbreviations: BMI, body mass index; AF, atrial fibrillation; CAD, coronary artery disease; CHF, congestive heart failure; HTN, hypertension; OSA, obstructive sleep apnea. Boxes (bivariate) and circles (multivariate) represent point estimates for linear regression coefficients, which correspond to the increase in average screen-time for a unit change in the corresponding variable. Whiskers give 95% confidence intervals. For categorical covariates (race/ethnicity, smoking, activity level) p values for the overall effect of the variable are presented. ^a^ Factors significantly associated with average screen-time at the p < 0.10 level in bivariate linear models were included in a multivariate linear model. ^b^ Education and income were both ascertained with 9 levels and analyzed as continuous variables. ^c^ PHQ-9 score is scaled to a unit increase of 5, the width of each category of depression. ^d^ Data were available on 267 participants. ^e^ White circles are regression coefficients adjusted for all other variables in the model.

In pursuing the hypothesis-free approach to identify predictors of screen-time, crude analyses revealed that younger participants, females, blacks, Hispanics, those self-reporting as “other” race, those of lower socioeconomic status (less education and lower income), those without a past history of smoking, those with a higher PHQ-9 score (more depressed mood), and those without atrial fibrillation, hyperlipidemia, or hypertension exhibited a longer overall average screen-time (Fig 3). After multivariate adjustment, only younger participants, blacks, and those reported to be of an “other” race demonstrated statistically significantly longer average screen-times (Fig 3). For instance, an individual 10 years older in age, on average after multivariable adjustment, had an average overall screen-time that was lesser by 0.7 minutes/hour (95% confidence interval 0.5 to 0.8, p < 0.001).

### Screen-Time and Sleep

**Fig 4:**
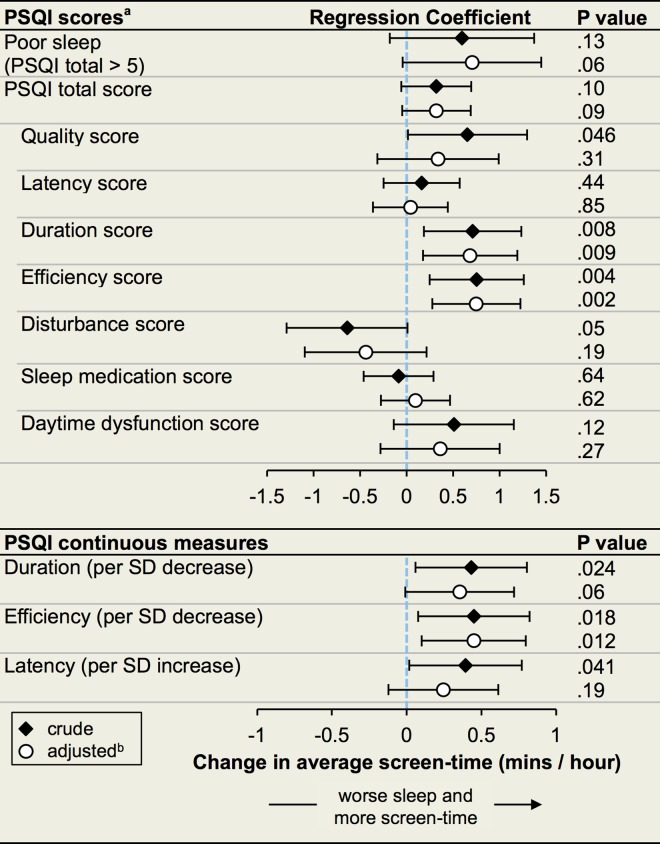
Associations between Baseline Sleep Quality and Average Screen-Time. Abbreviations: PSQI, Pittsburg Sleep Quality Index; SD, standard deviation. Diamonds (unadjusted) and circles (adjusted) represent point estimates for linear regression coefficients, which correspond to the increase in average screen-time for a unit change in the corresponding variable. Whiskers give 95% confidence intervals. Each PSQI score was analyzed as a continuous variable. Coefficients for PSQI total score are reported per SD increase, coefficients for Poor sleep and other PSQI component scores are reported per unit increase. ^a^ PSQI sub-scores range from 0 (good) to 3 (poor) for each component of sleep. The total score is the sum of the sub-scores (0–21). PSQI total score > 5 is a standard dichotomous measure for overall poor sleep. Decreased sleep duration and decreased sleep efficiency correspond to higher component scores. ^b^ Adjusted for age, sex, race/ethnicity, and history of obstructive sleep apnea.

In the hypothesis-driven analyses in the subgroup who also completed the sleep survey (n = 136), decreased sleep quality, shorter sleep duration, lower sleep efficiency, and longer sleep onset latency were each significantly associated with greater overall average screen-time in unadjusted analyses (Fig 4). After adjustment for age, sex, race/ethnicity, and history of sleep apnea, a greater score on the PSQI sleep duration component (less sleep) and reduced sleep efficiency were each statistically significantly associated with longer average screen-time (Fig 4).

**Fig 5:**
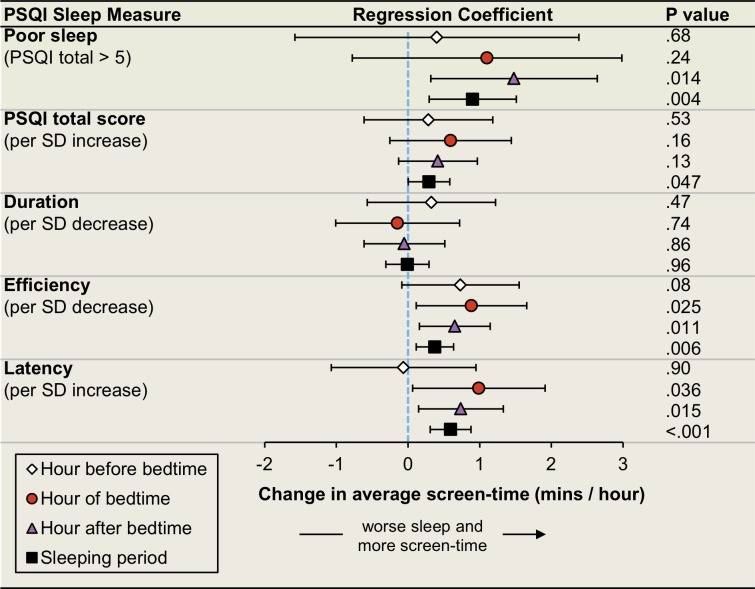
Associations between Baseline Sleep Quality and Average Screen-Time Within Sleep-Related Hours. Abbreviations: PSQI, Pittsburg Sleep Quality Index; SD, standard deviation. Among participants with a sleep survey and full screen-time data (N = 56), self-reported bedtime and wakeup-time was used to compute average screen-time (over 30 days) during the hour before bedtime, the hour of bedtime, the hour after bedtime, and during the sleeping period (all hours from bedtime to wakeup-time). All markers represent point estimates for linear regression coefficients after adjustment for age, sex, race/ethnicity, and history of obstructive sleep apnea. Coefficients correspond to the increase in average screen-time, during the indicated period, for a unit change in the corresponding sleep measure. Whiskers give 95% confidence intervals.

There were 56 participants that both completed the PSQI sleep survey and had complete screen-time data (0% missing). After adjustment for age, sex, race/ethnicity, and sleep apnea, poor sleep (by PSQI total > 5) was statistically significantly associated with longer average screen-time during the reported sleeping period and during the hour after bedtime (Fig 5). Both decreased sleep efficiency and increased sleep onset latency were associated with longer average screen-time during the reported sleeping period, the hour of bedtime, and the hour after bedtime. Total sleep duration was not associated with average screen-time during the reported sleeping period or during any of the 3 hours near reported bedtime.

## Discussion

Participants' smartphone screen-time exposure was on average approximately 3.7 minutes per hour, centered as expected primarily during the daytime. Those who were younger, Black, and Other race/ethnicity had greater screen-time exposure. Longer average screen-time was associated with shorter duration of sleep and reduced sleep efficiency. Given that screen-time after self-reported sleeping hours and near an individual’s bedtime was associated with reduced sleep efficiency and greater sleep onset latency, the relationship between overall smartphone use and sleep may be driven by exposure near bedtime.

Since the advent of the modern smartphone in 2007, the adoption and use of these devices has been rapid and widespread [13]. As of 2015, approximately 64% of all American adults owned a smartphone, including 85% of those aged 18 to 29 [1]. While the demographics of individuals that purchase smartphones has been studied, the frequency of use based upon an objective measure has not previously been described. We found that younger individuals on average have greater screen-time, in agreement with survey-based demographic and marketing research [1,13]. Our finding that African Americans and Hispanics exhibit longer screen-time is consistent with the fact that racial and ethnic minorities have fewer desktop and laptop computers at home and are more frequently dependent on their smartphone for internet access [1]. Of interest, neither income nor education was associated with average screen-time after multivariable adjustment, suggesting that something cultural aside from socio-economic status may be driving increased smartphone use in these populations.

While there has been concern for both the negative and positive impact of smartphones on depression [14,15], screen-time exposure was not associated with mood after multivariable adjustment. It is also noteworthy that unlike TV watching [16,17], smartphone screen-time was not associated with physical activity level or BMI. This may in part be due to the multi-functionality of smartphones, different modes of media consumption, or perhaps the use of smartphones during physical activities (i.e., running apps). Finally, despite the fact that searching for medical information is one of the most common activities carried out with smartphones [1], none of the medical conditions evaluated were associated with average screen-time.

In addition to how smartphones are changing modern life, the impact of screen exposure on sleep is a major concern. Poor sleep has been shown to decrease performance at school and work, is associated with depressed mood, and is established as a risk factor for obesity, cardiovascular disease, stroke, and death [4,18,19]. Smartphones are often nearby at night, and 68% of owners store their phone on a bedside table while they sleep [20]. Prior studies regarding watching television, conventional computer use, and video-games have demonstrated that use at nighttime and particularly in the bedroom is associated with poor sleep and insomnia [2,7]. Exposure to blue light from such backlit screens suppresses production of melatonin, thereby delaying sleep onset and reducing sleep duration and quality [21]. A recent randomized cross-over trail found hospitalized patients took longer to fall asleep and had reduced quality of sleep after reading a backlit eReader compared to a paper book before bedtime [22]. However, this and other experimental studies may not generalize to typical at-home screen exposure and experiences with eReaders may not extrapolate to smartphone use. At-home screen-time obtained by self-report has been associated with poor sleep [23], but a study that used an app to measure smartphone screen-time in a small group of Taiwanese university students found that participants underestimated their screen-time by approximately 9 hours per week (equivalent to an average of 3.2 minutes/hour) [24].

Our objectively measured screen-time was associated with reduced quality of sleep. That increased screen-time in the hour of and after bedtime, but not the hour before, was associated with greater sleep onset latency agrees with the notion that screen use just before attempting to fall asleep may be particularly problematic.

This study has several important limitations. First, we analyzed a self-selected group of participants that elected to enroll in the Health eHeart Study and download the Ginger.io app, which may not be representative of the general population. Compared to the U.S. census, the cohort is better educated, wealthier, and consists of a greater proportion of whites and females [25,26]. The relative older age of our cohort may be surprising in a study requiring smartphone ownership. However, as demonstrated by the standard deviations in age, the cohort generally represented those in their early 30s to those in their mid 60s. This likely demonstrates the age groups most representative not only of smartphone ownership, but also those most willing, interested, and able to engage with an internet-based research study. On the other hand, we could only record screen-time on Android phones, owners of which tend to have lower socio-economic status compared to iPhone owners [27,28]. Similarly, analyses on screen-time and sleep were limited to a subset who had both types of data available, and analyses on screen-time near bedtime were in a further subset with no missing screen-time data, both of which are susceptible to selection bias. Demographic, medical, and sleep information were obtained by self-report, although well-validated standardized scales (PSQI, PHQ-9, IPAQ) were used. In addition, self-report of medical conditions within the Health eHeart Study has previously proven to exhibit high accuracy [29]. App-recorded screen-time is subject to some measurement error. Screen-time when the app was actively turned off or the phone was in airplane mode was not captured, there could be multiple users of a single smartphone, or the screen could be on while a participant was not aware (such as when the screen is on while in a pocket). We also focused specifically on smartphone screen-time exposure and did not have information on the use of other back-lit devices (TV, computer, tablets). We analyzed 30-day windows with the least amount of missing screen data in an attempt to maximize the accuracy of the screen-time measure, but this could also have induced bias. Still, incomplete capture of smartphone screen exposure, and the absence of data on exposure to other types of screens, should only decrease sensitivity for associations with other participant characteristics. Finally, in regards to the analyses related to screen-time and sleep; although screen-time was collected prospectively and compared to sleep characteristics at baseline, we cannot exclude “effect-cause”—poor sleep could lead to more screen-time.

Our study also has some notable strengths. This is the first time smartphone screen-time exposure has been recorded prospectively and compared to demographic and medical information. Screen-time was measured unobtrusively during “at home” use over a 30 day period and thus is more comparable to every-day long term use than prior experimental studies. Furthermore, since enrollment and consent in the Health eHeart study is performed remotely, the cohort is not limited to a particular geographic location or clinical population.

Our findings suggest that smartphone screen-time is an important exposure associated with worse sleep. Since poor sleep has important health consequences, further investigation to determine the causal relationship between smartphone use and sleep is necessary. This study took advantage of an app to measure smartphone screen-time, but sleep habits were measured once via a survey. Future work may benefit from using an app to collect data on nightly sleep quality. A measure of overall screen-time from multiple devices would also be helpful in this regard. Since certain activities may be more stimulating (e.g. thumbing through Facebook posts), future work might explore how screen-time, smartphone activities, and sleep interact. Ultimately, a deeper understanding of the situational and cultural factors driving smartphone use will be needed to guide studies on interventions aimed at reducing screen-time to improve sleep.

## Conclusions

Our findings demonstrate that we spend a substantial portion of our time looking at our smartphones. Screen-time differs across age, race, and ethnicity and may be culturally driven by different norms or other environmental determinants. Screen-time exposure varies throughout the day, with most exposure occurring during the day, but some individuals have peaks of use during the night. Exposure to a smartphone screen, particularly around bedtime, is associated with a lower quality of sleep.

## Supporting Information

S1 File Copyright on Fig 1. Information on copyrights that apply to the map in Fig 1.
(PDF)
