# Supplementary material for: Direct Measurements of Smartphone Screen-Time: Relationships with Demographics and Sleep
Source: PLoS One. 2016 Nov 9;11(11):e0165331. doi: 10.1371/journal.pone.0165331 (PMC5102460; doi:10.1371/journal.pone.0165331)
Supplement: S1 File — Information on copyrights that apply to the map in Fig 1. (PDF) [file pone.0165331.s001.pdf]

Permissions for Figure 1

This figure was constructed using software provided by Tableau Incorporated (www.tableau.com) which uses a U.S. map provided under a CC BY-SA license from OpenStreetMap. An email exchanged between the authors and Tableau to request permission to use the figure is included below. The copyright from OpenStreetMap allows use with proper attribution. Further detail on the copyright from OpenStreetMap may be found at www.openstreetmap.org/copyright.

Email Exchange

Re: Requesting permission to publish an image created using Tableau software in a peer reviewed medical journal
Sarah Goehri [sgoehri@tableau.com]
Sent:Thursday, September 29, 2016 8:56 AM
To: Marcus, Greg; Public Relations [pr@tableau.com] Cc: Christensen, Matt

Hi Greg,

Thanks for reaching out. The visualizations are not proprietary to Tableau but we do appreciate the attribution of the software. We suggest the following statement:

Created with Tableau Software (www.tableau.com) and published with permission of the company.

Let us know if you have any additional questions and congrats on the success of your research!

Best,
Sarah

--
Sarah Goehri
Public RelaǺons Strategist Tableau SoĚware

t: 206.410.2079
c: 425.241.7172
e: sgoehri@tableau.com
